# Supplementary material for: The impact of climate change on flow conditions and wetland ecosystems in the Lower Biebrza River (Poland)
Source: PeerJ. 2020 Sep 11;8:e9778. doi: 10.7717/peerj.9778 (PMC7489244; doi:10.7717/peerj.9778)
Supplement: Supplemental Information 2 [file peerj-08-9778-s002.docx]

**SWAT model discharge time series used for trend analysis (Fig1-5).**

*
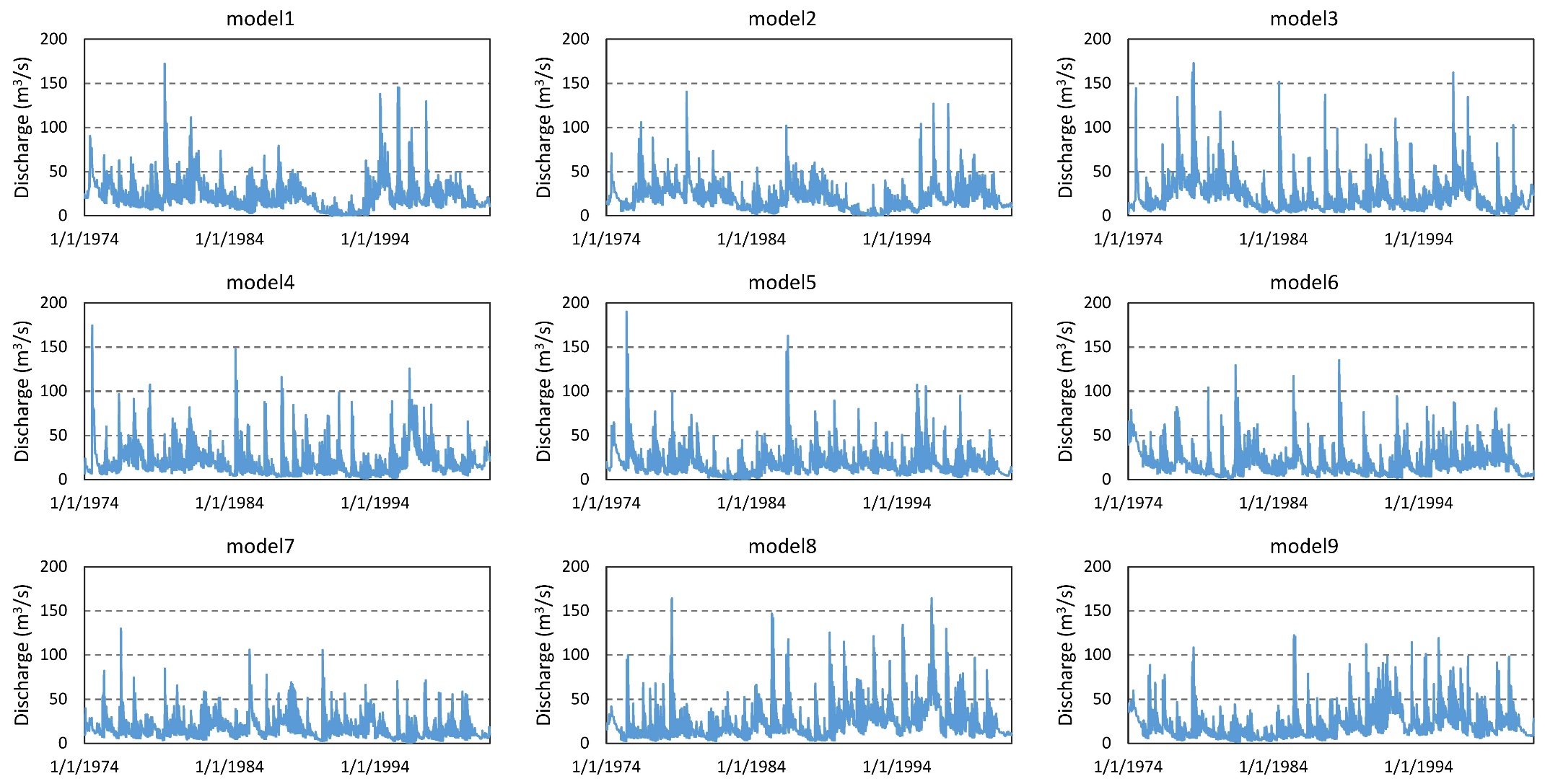
*

Figure 1 SWAT model simulated time series for 9 climate models for the historical period.


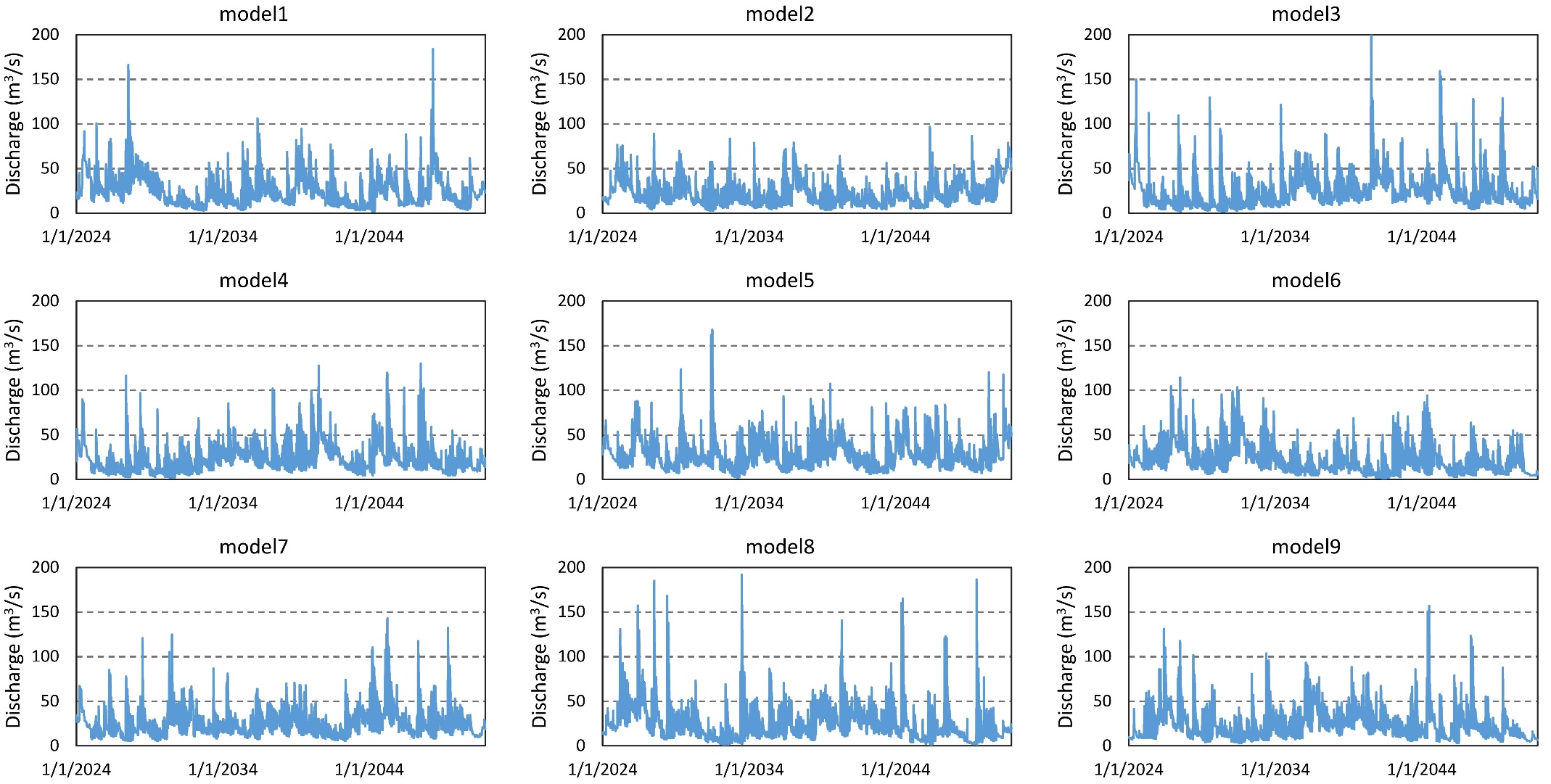


Figure 2 SWAT model simulated time series for 9 climate models for the near future under RCP4.5


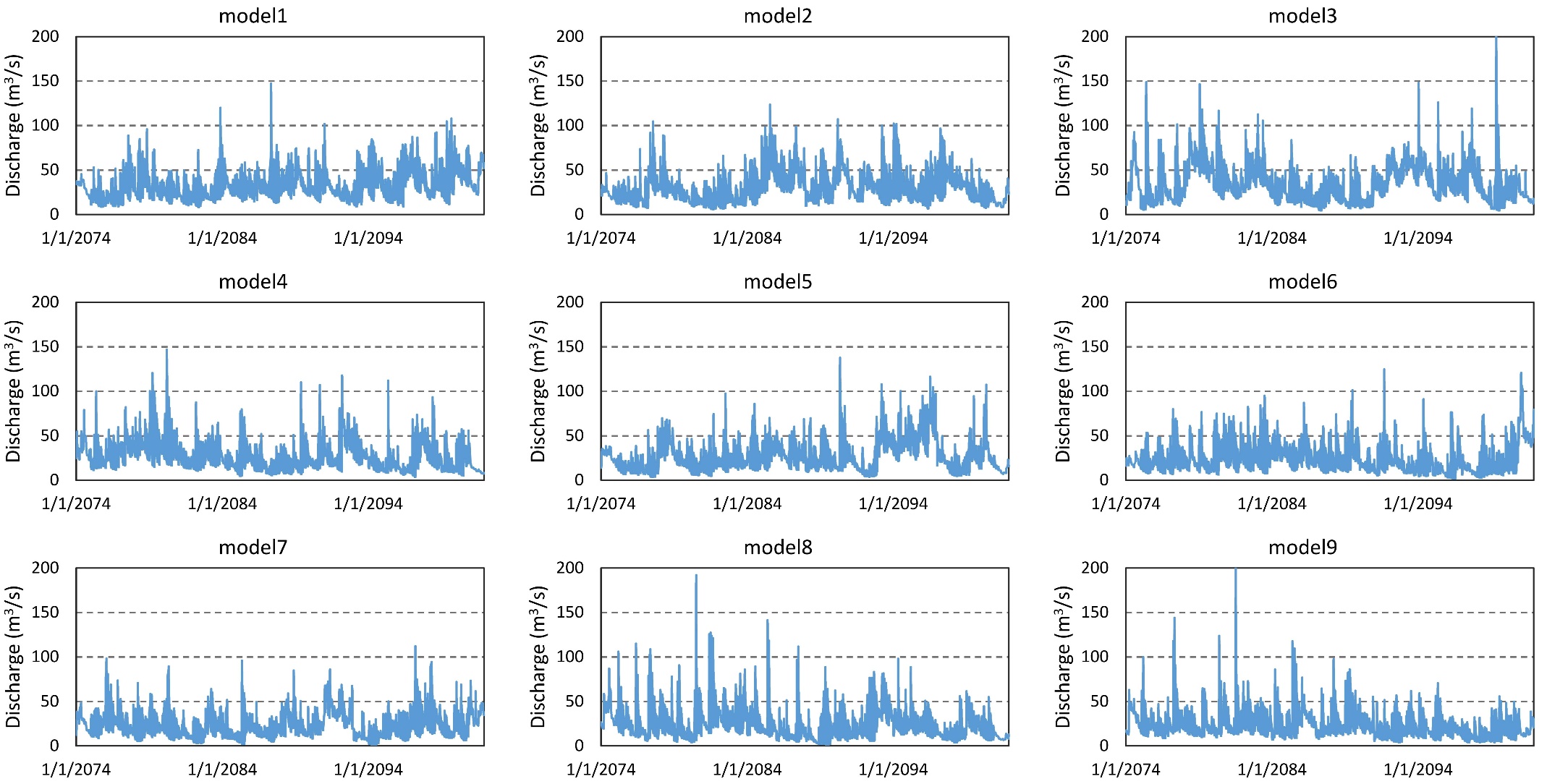


Figure 3 SWAT model simulated time series for 9 climate models for the far future under RCP4.5


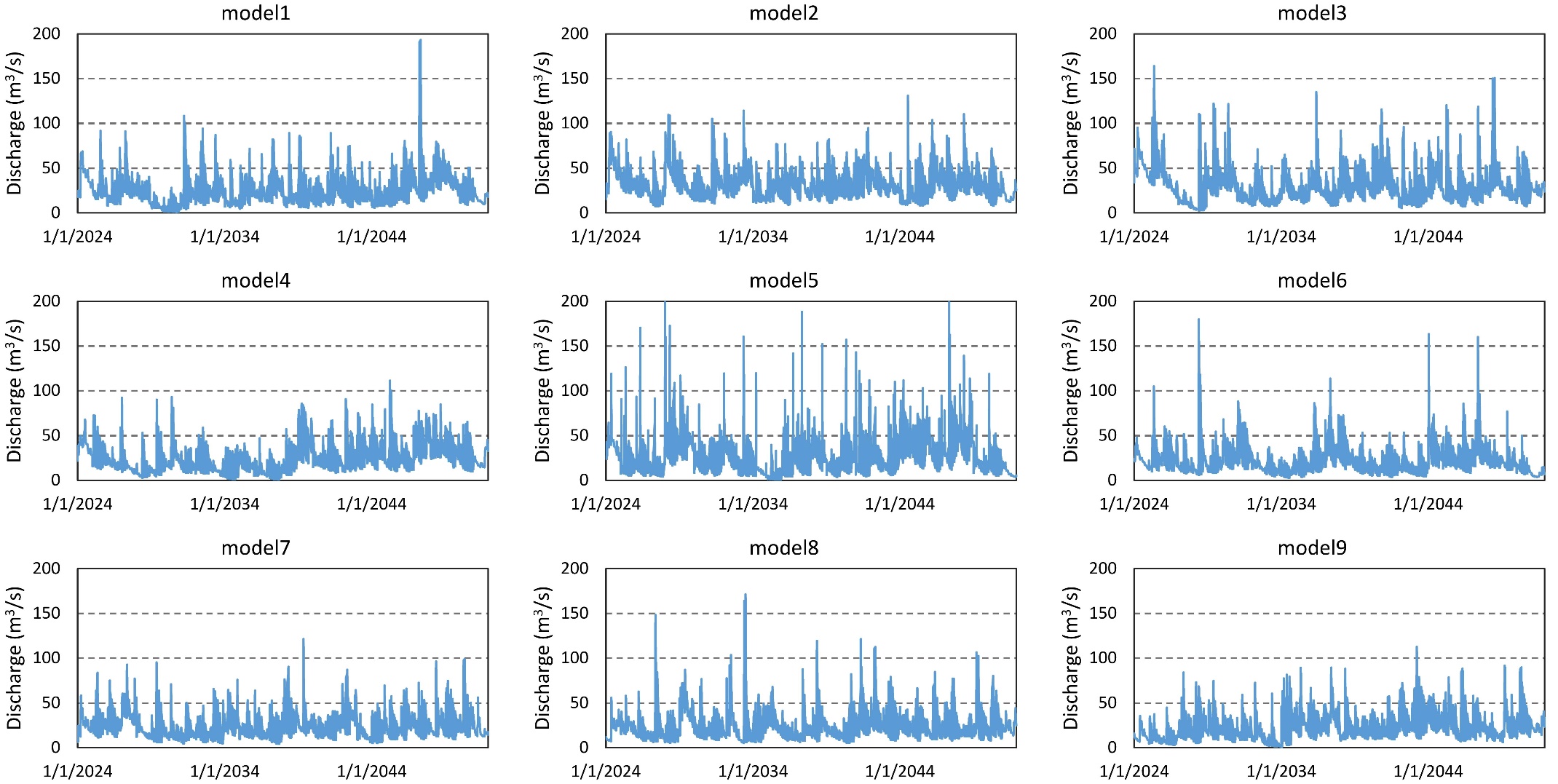


Figure 4 SWAT model simulated time series for 9 climate models for the near future under RCP8.5


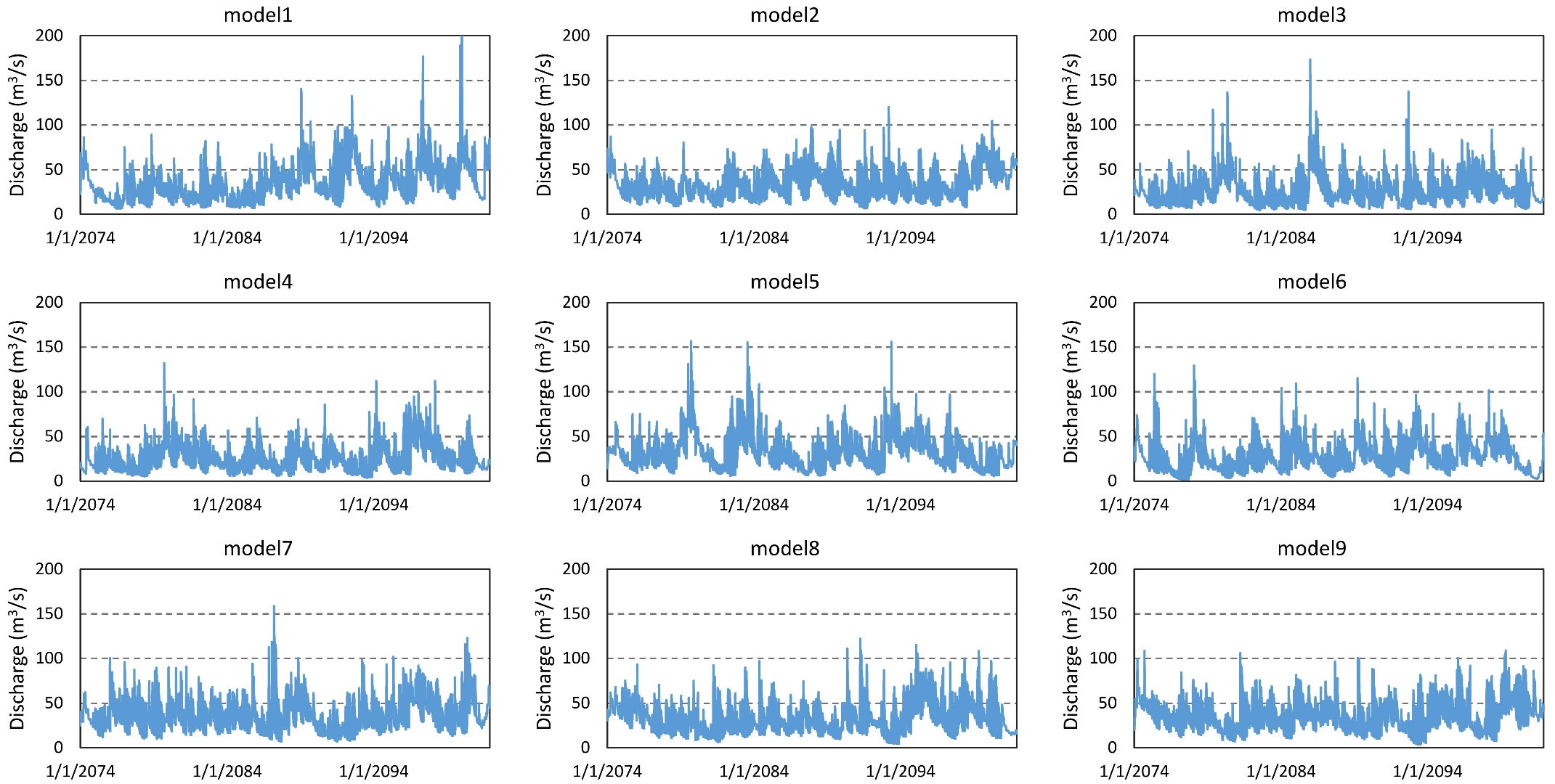


Figure 5 SWAT model simulated time series for 9 climate models for the far future under RCP8.5
